# OLFML2A Downregulation Inhibits Glioma Proliferation Through Suppression of Wnt/β-Catenin Signaling

**DOI:** 10.3389/fonc.2021.717917

**Published:** 2021-09-28

**Authors:** Shize Ma, Lei Duan, Huateng Dong, Xiaodong Ma, Xinyu Guo, Jianli Liu, Guoqiang Li, Yue Yu, Yanlong Xu, Guoqiang Yuan, Xingkun Zhao, Guopeng Tian, Shijia Zhai, Yawen Pan, Yinian Zhang

**Affiliations:** ^1^Department of Neurosurgery and Laboratory of Neurosurgery, Lanzhou University Second Hospital, Lanzhou, China; ^2^Second Clinical School, Lanzhou University, Lanzhou, China; ^3^Department of Pediatric Neurology, Gansu Provincial Maternity and Child-Care Hospital, Lanzhou, China; ^4^Department of Radiology, Lanzhou University Second Hospital, Lanzhou, China

**Keywords:** olfactomedin-like 2A (OLFML2A), glioma, amyloid precursor protein (APP), proliferation, apoptosis, Wnt/β-catenin signaling

## Abstract

Glioma is a highly heterogeneous and lethal tumor with an extremely poor prognosis. Through analysis of TCGA data, we identified that OLFML2A is a key promotor of gliomagenesis. However, the molecular function of OLFML2A and its underlying mechanism of action in glioma remain unclear. In this study, we found that OLFML2A expression was significantly upregulated in glioma specimens and positively correlated with pathological grades in glioma patients. Moreover, Kaplan–Meier survival analysis of TCGA data revealed that glioma patients with higher OLFML2A expression had shorter overall survival. Importantly, OLFML2A knockdown in glioma cells inhibited cell proliferation and promoted apoptosis. Mechanistically, OLFML2A downregulation inhibits Wnt/β-catenin signaling by upregulating amyloid precursor protein (APP) expression and reducing stabilized β-catenin levels, leading to the repression of MYC, CD44, and CSKN2A2 expression. Furthermore, OLFML2A downregulation suppressed the growth of transplanted glioma subcutaneously and intracranially by inhibiting Wnt/β-catenin pathway-dependent cell proliferation. By uncovering the oncogenic effects in human and rodent gliomas, our data support OLFML2A as a potential therapeutic target for glioma.

## Introduction

Glioma is characterized by extremely poor outcomes (1). In particular, glioblastoma (GBM), a World Health Organization (WHO) grade IV tumor, is the most common and aggressive malignant brain tumor in humans, and even with radical surgery, radiation therapy, and chemotherapy, the median survival time is less than 12 months (2, 3). Studies have partly characterized the genetic basis of tumorigenesis in glioma over the past two decades (4, 5). Based on these findings, the molecular classification standard of central nervous system tumors was established by the WHO in 2016 (6). However, glioma is a highly heterogeneous tumor (7), and there are many specific molecules that can be used for its classification. Thus, identifying the molecules that regulate glioma progression may contribute to the development of reliable therapeutic targets for glioma.

Olfactomedin-like 2A (OLFML2A) belongs to subfamily IV of olfactomedin domain-containing (OLFM) proteins (8), also known as photomedins-1, which were first identified and characterized in the mouse retina (9). In normal human tissue, OLFML2A is predominantly detected in the photoreceptor layer of the retina but is not expressed in neuronal tissues (10). In general, the function of OLFML2A remains poorly understood at present. Recent studies have shown that OLFML2A is highly expressed in triple-negative breast cancer (TNBC) and positively related to cell growth, proliferation, and migration (11). In acute myeloid leukemia, high OLFML2A expression is associated with extramedullary infiltration and predicts a poor prognosis (12). Consistent with these findings, Dai et al. showed that OLFML2A is also overexpressed in liver hepatocellular carcinoma (LIHC) and many other cancers, such as breast cancer and leukemia, by comparing the Oncomine dataset to normal samples (13). Ultimately, Kyoto Encyclopedia of Genes and Genomes (KEGG) pathway and process enrichment analysis suggested that OLFML2A regulates the tumorigenesis of LIHC through angiogenesis, negative regulation of DNA-binding transcription factor activity, regulation of neuronal differentiation, positive regulation of apoptosis, and autophagy *via* the Wnt, Notch, and hypoxia signaling pathways (13). The above observations underscore that OLFML2A may be involved in the development of many cancers. However, the detailed regulatory mechanisms have not yet been thoroughly validated, and the underlying molecular mechanisms need to be elucidated. Moreover, the role of OLFML2A in glioma has not been reported.

Therefore, to better understand the function of OLFML2A and identify new markers for targeted glioma therapy, *in vitro* and *in vivo* assays and bioinformatics analyses were performed to elucidate the function and mechanisms of OLFML2A in glioma progression.

## Materials and Methods

### Cell Culture

Human glioblastoma cell lines (U87MG and U251) were purchased from GeneChem (Shanghai, China) and cultured in DMEM (HyClone, USA) supplemented with 10% fetal bovine serum (Sigma, USA) and 1% penicillin and streptomycin (Beyotime). All cells were cultured in a humidified incubator at 37°C under an atmosphere with 5% CO_2_.

### Lentiviral Transduction

Cells were seeded in 6-well plates at a density of 5×10^4^ cells/well and incubated at 37°C until reaching 30% confluence. According to the determined multiplicity of infection, an appropriate amount of lentivirus was added to the culture medium of glioma cells for transduction, and the cells were cultured for another 14 h. Subsequently, the medium containing the lentivirus was removed, and the cells were cultured in normal medium for 58 h. GFP expression was observed under a fluorescence microscope 3 days after infection, and glioma cells with an infection efficiency of >80% were selected for subsequent analysis.

All lentiviruses were purchased from GeneChem (Shanghai, China). The forward sequences of the shRNAs targeting OLFML2A in U251 and U87MG cells were as follows: sh-OLFML2A-1 (TCTATGTCACCAACTACTA) and sh-OLFML2A-2 (GCCAAACAAACATTCACTA). The forward sequence of the shRNA targeting OLFML2A in C6 cells was as follows: AGGCCGGTGGAGTAATATGTA. To increase the efficiency of RNA interference, four shRNAs targeting amyloid precursor protein (APP) were designed, and the four plasmids carrying the different shRNAs were mixed in equal proportion for lentiviral packaging. The forward sequences of the shRNAs targeting APP in U87MG cells were as follows: psc10391 (ccCTGTTCATTGTAAGCACTT), psc10392 (gcAGACACAGACTATGCAGAT), psc11434 (ccCAAAGTTTACTCAAGACTA), and psc11435 (gcCATCTTTGACCGAAACGAA).

### Immunohistochemistry (IHC)

All glioma specimens were routinely embedded in paraffin and then analyzed by immunohistochemical staining. To this end, specimens were cut into 5-μm sections and then subjected to antigen retrieval. Subsequently, the sections were incubated with primary antibodies (anti-OLFML2A, Abcam, ab75882, 1:200; anti-Ki-67, Abcam, ab15580, 1:200; anti-APP, Abcam, ab32136, 1:300; anti-β-catenin, CST, CST#8480, 1:50; anti-P-β-catenin (Ser33/37/Thr41), CST, CST#9561, 1:200; anti-GSK-3β, CST, CST#12456, 1:800; and anti-P-GSK-3β, CST, CST#9323, 1:50) overnight at 4°C and then visualized using a DAB detection kit according to the manufacturer’s protocol. Then, the sections were counterstained with hematoxylin and observed under a BX-53 microscope (Olympus, Tokyo, Japan). The degree of immunostaining in the sections was reviewed and scored independently by two observers based on both the percentage of positively stained tumor cells and the staining intensity. The staining intensity was graded into four categories on a scale from 0 to 3 (intensity score): no staining (0), light-brown staining (1), brown staining (2), and dark-brown staining (3). Protein staining was evaluated using the following formula: overall staining score = intensity score × percentage score.

### Cell Proliferation Analysis by Celigo Assay

Cell proliferation was analyzed using a Celigo assay as previously described (14). Briefly, cells were cultured in 96-well plates at a density of 1,000 cells/well and treated with the indicated shRNAs or control shRNA (shCtrl). The medium was changed every other day, and the cells were imaged with a Celigo Imaging Cytometer for 5 consecutive days. For each experimental well of a 96-well plate, Celigo scans of four visual fields at each time point (40 × magnification) were performed, and the images were analyzed with the corresponding software. Each assay was conducted in triplicate. After 5 days of consecutive measurements, a cell growth curve was plotted to assess cell proliferation.

### 3-(4,5-Dimethylthiazol-2-yl)-2,5-Diphenyl Tetrazolium Bromide (MTT) Assay

MTT and dimethyl sulfoxide (DMSO) solutions (Sigma-Aldrich, St. Louis, MO, USA) were used to assess the cell proliferation. Specific cells (2×10^4^) were seeded into 96-well plates, and sterile MTT solution (10 μl) was added to each well after for 24, 48, and 72 h of cultivation. After culturing for 4 h at 37°C, the medium was removed, and 150 μl DMSO was added to each well. Then, the absorbance at 490 nm was measured with a microplate reader (SpectraMax i3X, MOLECULAR, CA, USA).

### Quantitation of Apoptosis by Flow Cytometry

An Annexin-V-APC apoptosis detection kit (eBioscience, Cat. No. 88-8007) was used to evaluate cell apoptosis according to the manufacturer’s instructions. First, glioma cells were infected with a lentivirus expressing OLFML2A or a scrambled shRNA sequence (Scr-shRNA). Subsequently, after culturing for another 4 days, the cells were harvested, washed with PBS, and then resuspended in staining buffer at a final density of 1×10^6^-1×10^7^/ml. Then, 5 μl of Annexin-V-APC was added to 100-μl aliquots of cell suspensions and incubated at room temperature for 10–15 min. Signals were detected with a FACSCalibur instrument (Becton-Dickinson, USA).

### Animal Studies

All animals were purchased from the Animal Experiment Center of Gansu College of Traditional Chinese Medicine (Lanzhou, China) and housed in a standardized specific pathogen-free animal facility. All experimental procedures were approved by the Institutional Animal Care and Use Committee of Lanzhou University Second Hospital. All mice were maintained and were used in accordance with the guidelines approved by the Institutional Animal Care and Use Committee.

For the tumor xenograft implantation model, 4-week-old mice were randomly divided into two groups (n=10 per group) and implanted with 1×10^6^ U87MG cells that were previously transfected with OLFML2A shRNA lentivirus or the negative control. Then, the cells were mixed with Matrigel (50% volume) and subcutaneously implanted into the right flanks of the nude mice (BALB/c). Tumor volume was determined using an external caliper every 3–4 days and calculated using the formula (Length×Width^2^)/2. Mice were sacrificed 50 days after transplantation, at which time tumors were excised and used in subsequent analyses.

For the orthotopic model, 1×10^6^ C6-OLFML2A-shRNA or control cells were injected into the right corpus striatum of the brains of 6-week-old Wistar rats using a stereotactic frame (n=5 per group). Spectral computed tomography (HD750 CT scanner; GE Healthcare, Little Chalfont, UK) was performed to record tumor growth at 14 and 19 days after injection using the same scanning parameters described in a previous study (15). Rats were monitored and sacrificed when neurological signs appeared or after 35 days.

### Gene Chip Microarray Analysis

Total RNA was extracted using an RNAiso Plus kit (TaKaRa Biotechnology Co., Ltd., Dalian, China) and assessed using a NanoDrop 2000 Spectrophotometer (Thermo Scientific, MA, USA) and an Agilent Bioanalyzer 2100 (Palo Alto, CA, USA) (16). Only samples of sufficient quality were used in subsequent gene chip experiments. The RNA quality control standards were as follows: 1.7<A260/A280<2.2 on the NanoDrop 2000 and an RNA integrity number (RIN)> =7.0 and a 28S/18S ratio > 0.7 on the Agilent 2100 Bioanalyzer. The microarray was processed as described in a previous study (17). Briefly, amplified RNA (aRNA) was prepared from total RNA using a GeneChip 3’ IVT Express kit (Affymetrix, Santa Clara, CA, USA). Then, DNA was synthesized, after which double-stranded template DNA was synthesized. Biotin-labeled aRNA was obtained by *in vitro* transcription. Then, aRNA was purified, fragmented, labeled, and hybridized with an Affymetrix GeneChip^®^ Human Transcriptome Array 2.0 (Affymetrix, Santa Clara, CA, USA). After hybridization, the chips were washed and stained. Finally, raw intensity data were analyzed using Affymetrix Expression Console software after the images and raw data were scanned. Genes that were up- or downregulated with fold changes of > 2.0 or < 2.0 were analyzed using Affymetrix Transcriptome Analysis Console Software.

### Ingenuity Pathway Analysis

Ingenuity pathway analysis (IPA) of differentially expressed genes (DEGs) was conducted with Qiagen’s Ingenuity Pathway Analysis algorithm (www.qiagen.com/ingenuity, Qiagen, Redwood City, CA, USA). Canonical pathway analysis, functional analysis, regulatory effect analysis, and interaction network analysis were performed, with activation z-scores and overlapping p-values calculated as described in a previous study (18).

### Quantitative Reverse Transcription PCR

Cells in the exponential growth phase were collected and lysed with an RNAiso Plus kit (TaKaRa Biotechnology Co., Ltd., Dalian, China) to extract total RNA. A NanoDrop-2000 spectrophotometer (Thermo Scientific, MA, USA) was used to measure the purity and concentration of RNA. According to the manufacturer’s protocol, total RNA was reverse-transcribed using the Prime Script RT kit (TaKaRa Biotechnology Co., Ltd.) for 15 min at 37°C before being heat inactivated for 20 s at 85°C. Subsequently, quantitative reverse transcription PCR (qRT-PCR) was performed using SYBR^®^ Premix Ex Ta Master Mix (TaKaRa Biotechnology Co., Ltd.) and a Bio-Rad CFX96 Real-time PCR system (Bio-Rad Laboratories, Inc., Hercules, CA, USA). The primers used for qRT-PCR are shown in **Table 1**. qRT-PCR was performed in a final volume of 10 μl with the following thermocycling program: an initial denaturation at 95°C for 10 min followed by 40 cycles of 95°C for 10 s, 60°C for 30 s, and 72°C for 30 s. As the reaction progressed, fluorescence signals were obtained. After amplification, a melting curve analysis was performed, the results of which were used to determine the dissociation characteristics of the PCR products. The 2^-ΔΔCq^ method was used to calculate the mRNA expression level relative to that of the internal reference gene GAPDH. Each sample was assayed in triplicate.

**Table 1:** Primer sequences used in this study.

Gene	Forward primer 5’-3’	Reverse primer 5’-3’
*GAPDH*	*TGACTTCAACAGCGACACCCA*	*CACCCTGTTGCTGTAGCCAAA*
*OLFML2A*	*AACAGGCAGTAGAGTCAA*	*TTACAAGATTCCTACCAACAG*
*APP*	*CTGATGCGGAGGAGGATGAC*	*TCTCTGTGGCTTCTTCGTAGG*
*Wnt3*	*GGACGGAGAAGCGGAAGGA*	*GCGAGTTGGGTCTGGGTCAT*
*Wnt5a*	*TCGACTATGGCTACCGCTTTG*	*CACTCTCGTAGGAGCCCTTG*
*Wnt5b*	*CTGGTGGTCATTAGCTTTG*	*ATGTGCTCCTGGTACAATT*
*MYC*	*TGTCCGTCCAAGCAGAGG*	*CGCACAAGAGTTCCGTAGC*
*CD44*	*AGGCTGAGACAGGAGGTTA*	*CCTCCCTTATTTCTATCGTG*
*CSNK2A2*	*TGGAGTTTGGGCTGTATGTT*	*TCGTATCGCAGAAGTTTGTC*
*LEF1*	*AGAGCGAATGTCGTTGCTGA*	*TCGTTTTCCACCTGATGCAGA*
*FZD3*	*CAAATCTGGGTGTTGGGTT*	*TGAGAAAGGCTGGGCATC*
*LRP6*	*TGGGGAAACTATGACTAATG*	*CTGACAAAGAACTTGGGTG*
*LRP1*	*AGGGCGTAGGTTCCTTTCTC*	*CATTGGTCACCACGTCTTCA*
*DVL3*	*TACTGCGGGAGATTGTGC*	*GAACTGGTGATGGAGGAGC*
*SOX4*	*ACTTCGAGTTCCCGGACTACT*	*TGAAAACCAGGTTGGAGATGC*
*MAP3K7*	*CCGGTGAGATGATCGAAGCC*	*GCCGAAGCTCTACAATAAACGC*
*AKT3*	*AATGGACAGAAGCTATCCAGGC*	*TGATGGGTTGTAGAGGCATCC*
*MDM2*	*GAATCATCGGACTCAGGTACATC*	*TCTGTCTCACTAATTGCTCTCCT*
*RARG*	*ATGCTGCGTATCTGCACAAG*	*AGGCAAAGACAAGGTCTGTGA*
*BTRC*	*AGTTCTGCACTTGCGTTTC*	*ACTCACTACCAGCCTGTCC*
*TGFBR2*	*GTGCCAACAACATCAACC*	*GACTGCCACTGTCTCAAACT*
*ACVR1C*	*GCACCTTCCAACAGCATCAC*	*ATCCAGAGGCGGTCACATC*
*ACVR2B*	*AGACACGGGAGTGCATCTACT*	*GCCTATCGTAGCAGTTGAAGTC*
*PPP2R2B*	*ACCAGGGACTACTTGACCG*	*TCACGCTTGGTGTTTCTGT*

### Western Blot Analysis

Cells were lysed using RIPA buffer (Beyotime Institute of Biotechnology) on ice for 30 min, after which the samples were centrifuged at 12,000×g for 45 min at 4°C. The protein concentrations of the samples were determined using a BCA protein assay kit (Solarbio, China), after which the proteins were subjected to SDS-PAGE and transferred to PVDF membranes. The membranes were then blocked with TBST containing 5% bovine serum albumin (BSA; Solarbio, China) for 2 h at room temperature and then incubated with primary antibodies against the following antigens: OLFML2A (1:500, Abcam, ab85458, UK); GAPDH (1:1,000, Abcam, ab8245, UK); APP (1:500, Abcam, ab32136, UK); β-catenin (1:500, CST, CST#8480, USA); P-β-catenin (Ser33/37/Thr41) (1:500, CST, CST#9561, USA); GSK-3β (1:500, CST, CST#12456, USA); and P-GSK-3β (1:500, CST, CST#9323, USA) overnight at 4°C. The membranes were then washed three times with TBST and incubated with a horseradish peroxidase-conjugated secondary antibody for 2 h at room temperature. The blots were visualized with an enhanced chemiluminescence (ECL) kit (Solarbio, China) and scanned with ChemImager 5500 V2.03 software. The relative integrated density values (IDVs) were calculated using Fluor Chen 2.0 software using GAPDH as an internal control.

### Statistics and Data Analysis

All data are presented as means ± standard deviation (SD). Statistical analyses were performed using Prism 8 (GraphPad Software, San Diego, CA, USA). Two-tailed Student’s t-test was used to assess the significance of the differences between two groups, and one-way ANOVA was used to assess the significance of the differences among multiple groups. Survival curves were analyzed using the Kaplan–Meier method and assessed by the log-rank test. P-values<0.05 were considered to indicate a significant difference.

## Results

### OLFML2A Overexpression Is Correlated With Glioma Progression and Poor Prognosis

**Figure 1:**
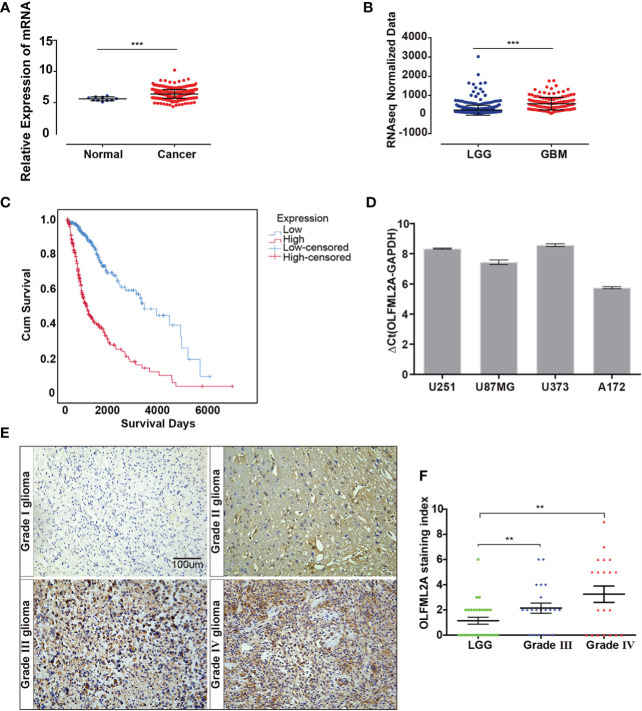
OLFML2A expression is elevated in gliomas and correlated with tumor grade and prognosis. **(A)** Expression profile of OLFML2A mRNA in primary glioma tissues (n=548) and normal brain tissues (n=10; Mann–Whitney U test; ***P < 0.001; TCGA). **(B)** Expression profile of OLFML2A mRNA in low-grade glioma (LGG) samples (n=515) and glioblastoma (GBM) samples (n=152; Mann–Whitney U test; ***P < 0.001; TCGA). **(C)** Kaplan–Meier survival curves comparing glioma patients with low and high OLFML2A expression levels (n = 664; P < 0.001; TCGA) based on the median value of OLFML2A mRNA. **(D)** Real-time PCR analysis of OLFML2A expression in four human glioma cell lines. **(E, F)**. Immunohistochemistry (IHC) analysis of OLFML2A in 69 specimens with low- and high-grade gliomas. Scale bar=100 μm. The score was calculated according to the degree of cell staining and the proportion of positive cells. The staining index is shown as the product value of two scores. (**P < 0.01).

By analyzing datasets from The Cancer Genome Atlas (TCGA), we observed that OLFML2A expression was significantly upregulated in glioma tissues compared to normal brain tissue and that OLFML2A expression was positively correlated with glioma grades (**Figures 1A, B**). Kaplan–Meier survival analysis of TCGA data revealed that glioma patients with higher OLFML2A expression had shorter overall survival (**Figure 1C**). Real-time PCR results showed that OLFML2A expression was upregulated in four different glioma cell lines (**Figure 1D**). Taken together, these findings suggested that OLFML2A is overexpressed in human glioma.

To determine the clinical relevance of OLFML2A in glioma patients, OLFML2A expression was examined in 69 paraffin-embedded, archived glioma tissues by IHC. As expected, OLFML2A was significantly upregulated in glioma tissues (**Figure 1E**). Furthermore, OLFML2A levels were positively correlated with pathological grades in glioma patients (**Figure 1F**). Collectively, these findings confirmed the TCGA analysis results and suggested a potential association between OLFML2A upregulation and the progression of glioma.

### OLFML2A Expression Is Efficiently Inhibited by Lentiviral-Based shRNA in Human Glioma Cell Lines

**Figure 2:**
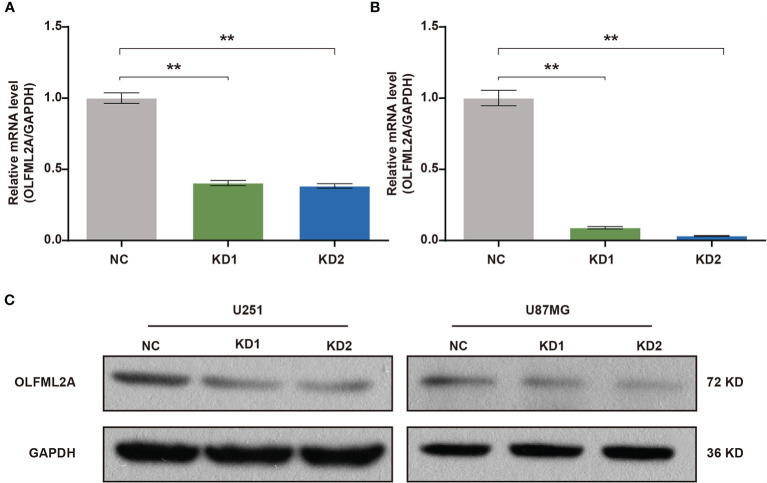
OLFML2A expression is efficiently inhibited by lentiviral-based shRNA in human glioma cell lines. **(A, B)** OLFML2A mRNA levels were assessed by qRT-PCR in U251 **(A)** and U87MG **(B)** cells. **(C)** OLFML2A protein content in glioma cell lines was assessed by western blotting. **P < 0.01. NC, cells infected with negative control lentivirus expressing Scr-shRNA; KD1 and KD2, cells infected with an OLFML2A-knockdown lentivirus expressing OLFML2A-shRNA-1 and OLFML2A-shRNA-2, respectively.

A positive correlation between OLFML2A expression and glioma pathological grades suggested that OLFML2A may be involved in the development and progression of glioma. To assess the biological role of OLFML2A in glioma, we used a lentivirus-based shRNA strategy to knockdown OLFML2A expression in glioma cell lines. U251 and U87MG cells were infected with a lentivirus specifically targeting human OLFML2A (OLFML2A-shrNA) or a negative control (Scr-shRNA) to evaluate the knockdown efficiency. Cells infected with the lentivirus were cultured and harvested, and total RNA/protein samples were collected. Then, the OLFML2A mRNA and protein levels were detected by qRT-PCR and Western blotting. The results demonstrated that OLFML2A mRNA expression was significantly reduced (**Figure 2A**), and OLFML2A protein was barely detectable in cells treated with OLFML2A-shRNA (**Figures 2B, C**). These results indicated that lentivirus-based shRNA strategy could inhibit OLFML2A expression at the protein and mRNA levels.

### OLFML2A Knockdown Leads to Reduced Glioma Cell Proliferation *In Vitro*

**Figure 3:**
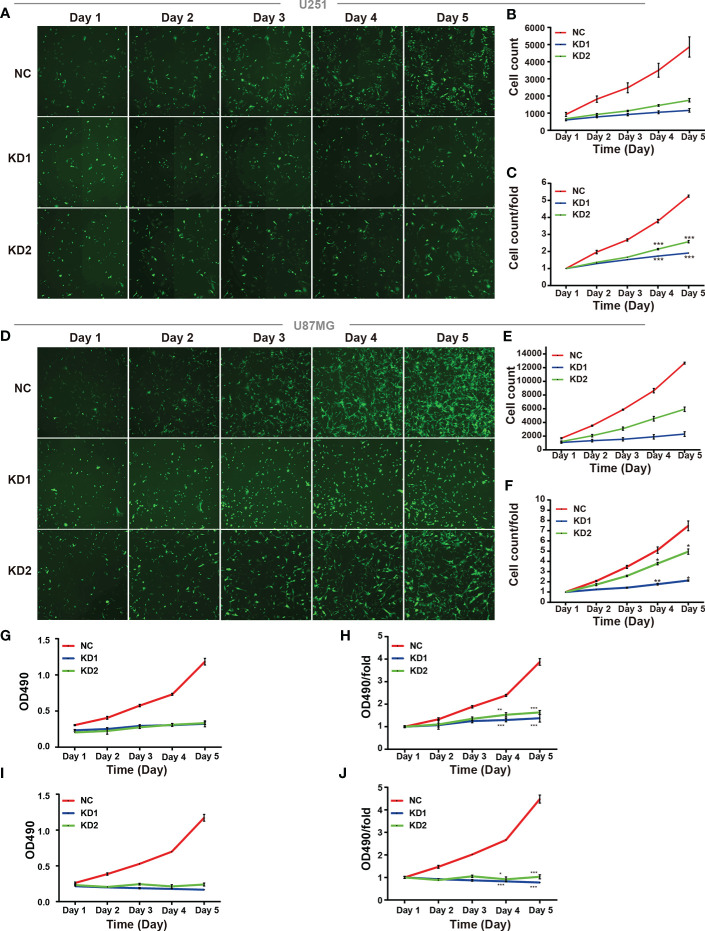
OLFML2A knockdown inhibits cell growth and proliferation. **(A–F)** U251 and U87MG cell growth was measured by Celigo assays for 5 days. **(G–J)** U251 and U87MG cell growth was measured by the MTT assay for 5 days. The data are presented as mean ± SD. *P < 0.05, **P < 0.01, ***P < 0.001.

U251 and U87MG cells express high levels of OLFML2A protein. To determine whether OLFML2A is required for the proliferation of these cell lines, we generated U251 and U87MG cells with lentivirus-delivered OLFML2A shRNA knockdown as previously described. Then, Celigo assays were performed to monitor cell growth for 5 days. As shown in **Figures 3A–F**, silencing OLFML2A decreased the total cell numbers and slowed the growth rate of U251 and U87MG cells. Subsequently, the MTT assay was used to further evaluate the effect of OLFML2A knockdown on the proliferation of U251 and U87MG cells. As indicated in **Figures 3G–J**, the cell proliferation rate of the OLFML2A-shRNA group was markedly reduced compared to that of the control group.

### Suppression of OLFML2A Expression Leads to Increased Glioma Cell Apoptosis

**Figure 4:**
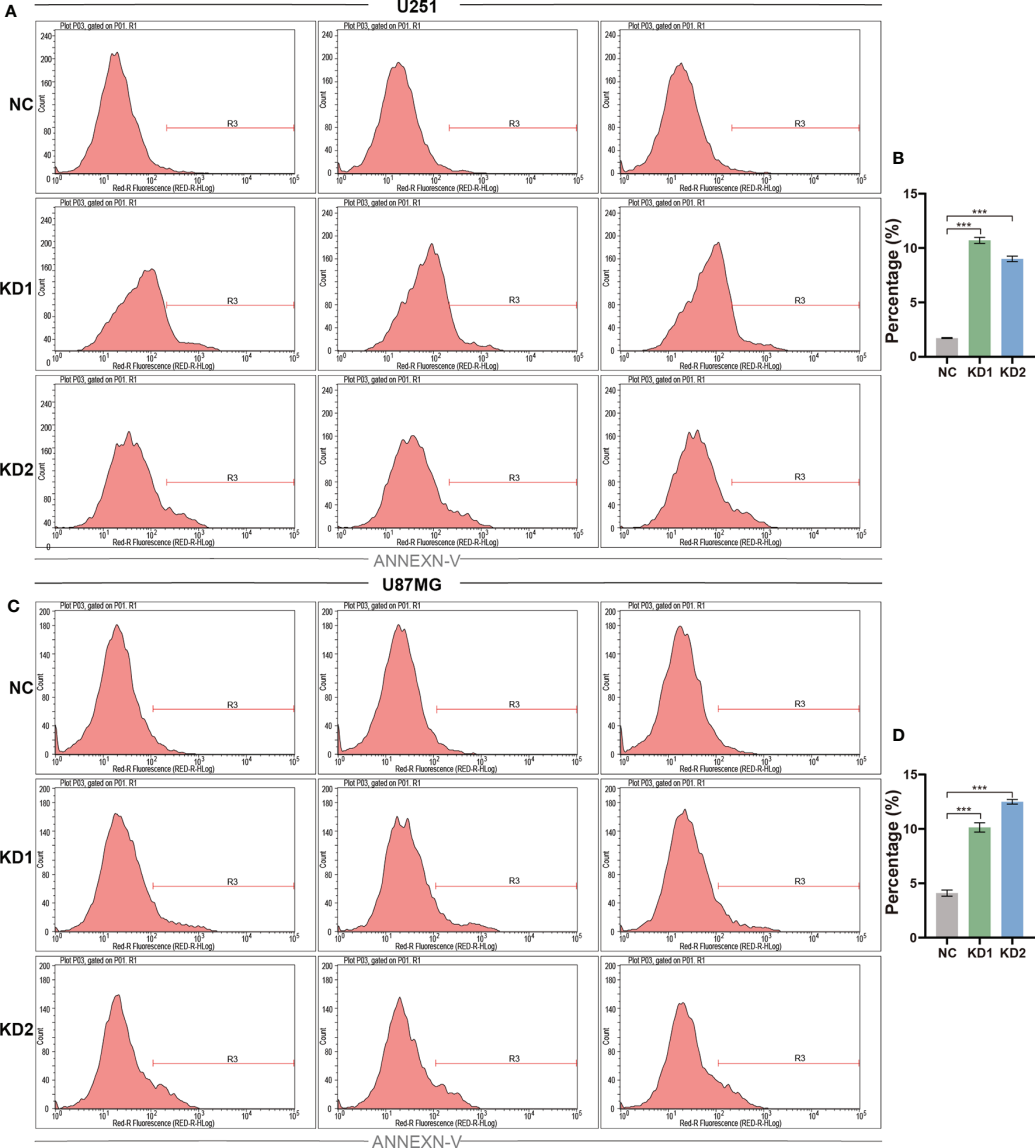
OLFML2A suppression promotes the apoptosis of glioma cells. **(A–D)** U251 and U87MG cell apoptosis was assessed using Annexin-V staining and analyzed by flow cytometry. The rate of apoptosis is presented as the mean ± SD. ***P < 0.01.

The ability to resist cell death, another hallmark of cancers, is essential for tumor development (19). The reduction in cell numbers induced by OLFML2A knockdown may be attributable to cell proliferation inhibition and increased cell apoptosis. To further investigate whether OLFML2A promotes glioma cell proliferation by regulating cell apoptosis, we used Annexin-V staining to assess apoptosis in OLFML2A-knockdown and control U251 cells. OLFML2A knockdown significantly induced apoptosis in U251 cells (**Figures 4A, B**), and similar results were observed in U87MG cells following OLFML2A silencing by shRNA (**Figures 4C, D**).

### Disruption of Multiple Crucial Cell Cycle Regulation Pathways Involved in Cancer Development by OLFML2A Knockdown

**Figure 5:**
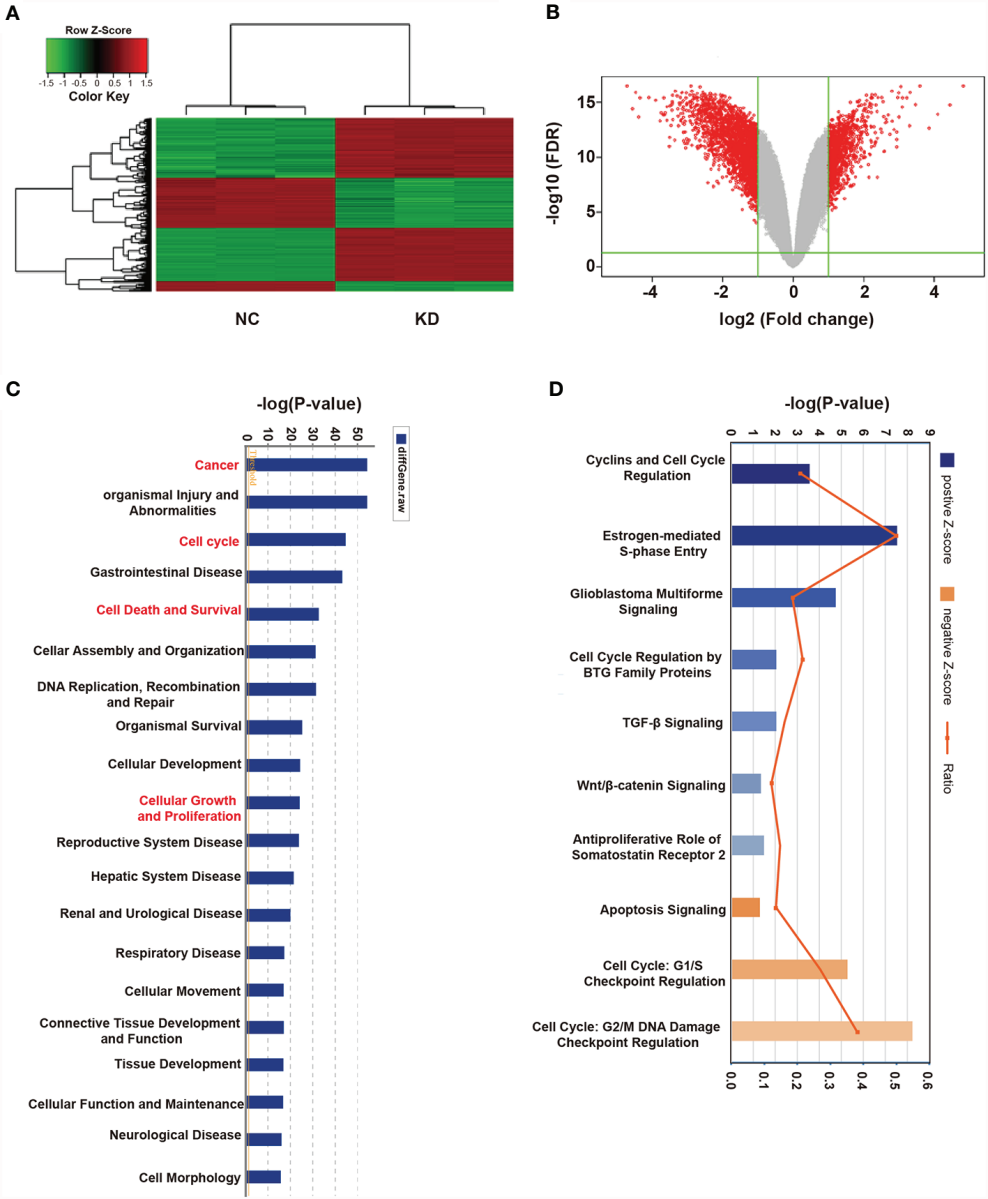
Widespread changes in gene expression in U87MG cells with OLFML2A knockdown as determined by microarray analysis. **(A)** Heatmap representation of 1,911 genes showing significant differential expression in the human malignant glioma cell line U87MG infected with a lentivirus expressing either Scr-shRNA (NC) or OLFML2A-shRNA (KD) under the criteria P<0.05 and |fold change|>2. Genes and samples are listed in rows and columns, respectively. The color scale for the normalized expression data is shown at the bottom of the microarray heatmap (green and red represent down- and upregulated genes, respectively). **(B)** Volcano plots. The genes with significantly altered expression are marked in red. The selected thresholds were a |fold change| > 2 and P < 0.05. **(C)** Disease and function analysis was performed to classify the enriched genes after OLFML2A silencing. **(D)** Annotated classical pathway analysis indicated that multiple signaling pathways involved in cell proliferation were enriched after OLFML2A silencing. Blue and orange represent suppressed and activated signaling pathways, respectively, and the intensity of the color indicates the degree of activation or inhibition. NC, cells infected with negative control lentivirus Scr-shRNA; KD, cells infected with OLFML2A-shRNA-1.

The results described above showed that OLFML2A is crucial for tumor development in human glioma cell lines. However, the mechanisms underlying OLFML2A-mediated glioma development and its downstream pathways have not been systematically explored. Therefore, global gene expression profiling of U87MG cells infected with lentiviruses expressing either Scr-shRNA or OLFML2A-shRNA was performed using a microarray platform, and 1,911 genes showing significant differential expression were identified (based on |fold change|≥2.0 and FDR<0.05), of which 658 were upregulated and 1,253 were downregulated (**Figures 5A, B** and **Supplementary Table 1****,** **Supplementary Data Sheet 1**). Then, functional analysis was performed *via* IPA, and the DEGs were observed to be enriched in cancer, cell cycle, cell death and cell survival, cellular growth, and proliferation, suggesting that OLFML2A is significantly (based on a P<0.001 threshold) involved in proliferation and cell cycle regulation processes (**Figure 5C**).

Canonical pathway analysis using IPA was conducted to further evaluate potential signaling pathways, resulting in 136 pathways being identified (**Supplementary Table 2**-Canonical Pathways.xlsx), of which 117 were inhibited and 19 were activated following OLFML2A gene suppression (based on |Z-score|>0 and P<0.05). Notably, among the inhibited signaling pathways, those involved in cell proliferation and cell cycle regulation, such as Cyclins and Cell Cycle Regulation, Estrogen-mediated S-phase Entry, and Glioblastoma Multiforme Signaling, were significantly inhibited (based on |Z-score|>2 and P<0.05) (**Figure 4D**). In addition, Cell Cycle Regulation by BTG Family Proteins (Z-score=-1.633), TGF-β Signaling (Z-score=-1.387), Wnt/β-catenin Signaling (Z-score=-1.342), and Antiproliferative Role of Somatostatin Receptor 2 (Z-score=-1.265), which are important signaling pathways in glioma proliferation and cell cycle regulation, were also inhibited (**Figure 4D**). Furthermore, Apoptosis Signaling (Z-score=1.508), Cell Cycle: G1/S Checkpoint Regulation (Z-score=1.291), and Cell Cycle: G2/M DNA Damage Checkpoint Regulation (Z-score=0.943) were activated (**Figure 5D**). Collectively, these findings provided a strong rationale for the role of OLFML2A as a crucial molecule that regulates the proliferation of glioma cells.

### OLFML2A Knockdown Suppresses the Wnt/β-Catenin Pathway in Glioma Cells

**Figure 6:**
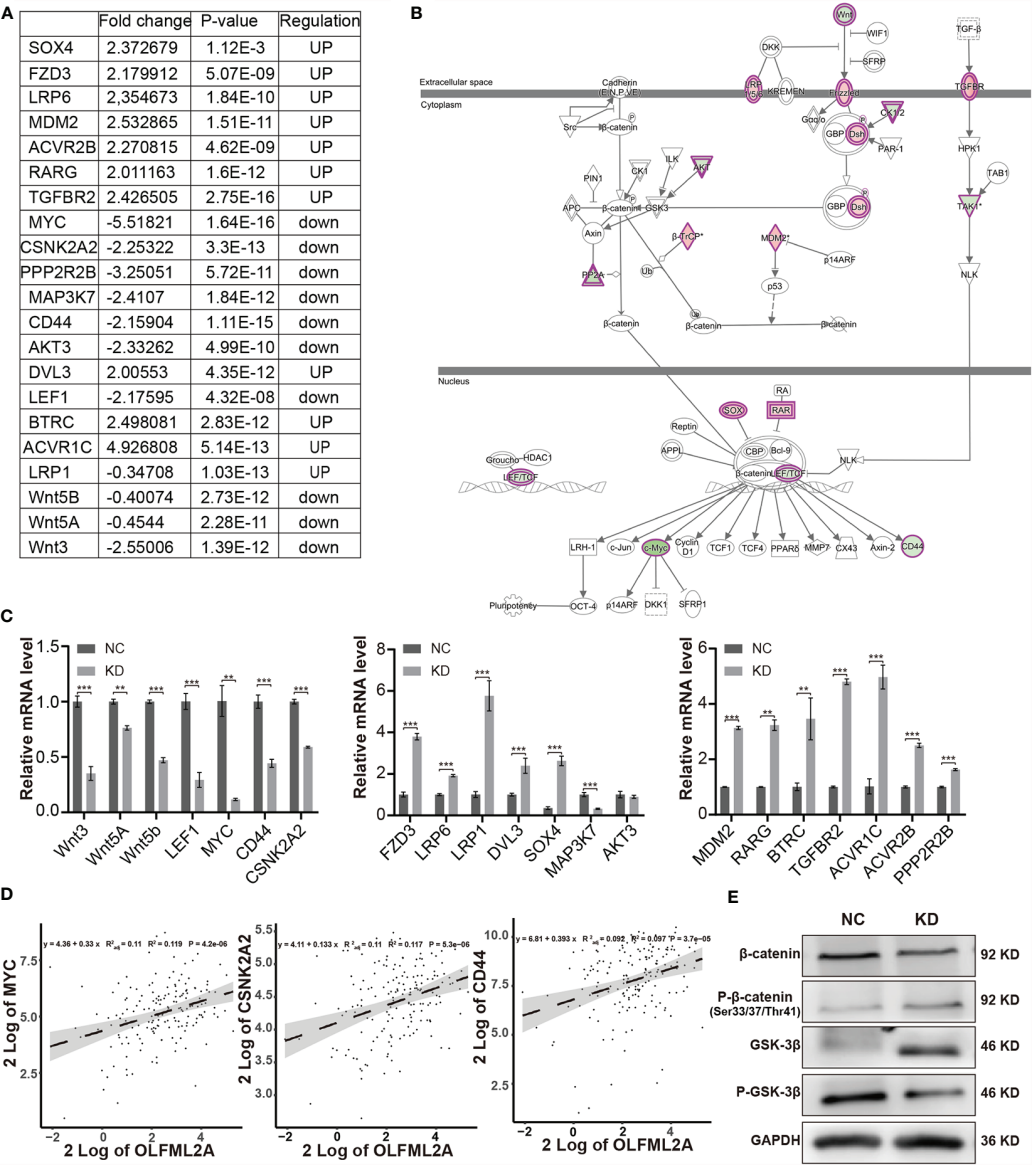
OLFML2A knockdown suppresses the Wnt/β-catenin pathway in glioma cells. **(A)** The fold change, P-values, and regulatory effect of the molecules associated with the Wnt/β-catenin signaling pathway relative to OLFML2A knockdown are collated in the table. **(B)** The Wnt/β-catenin signaling pathway was mapped by IPA, and the 21 molecules involved in the Wnt/β-catenin pathway are included in the table **(A)**. Green: downregulated. Red: upregulated. **(C)** The expression of the 21 molecules involved in the Wnt/β-catenin pathway was verified by qRT-PCR in U87MG cells. **P < 0.01, ***P < 0.001. **(D)** The correlation of OLFML2A expression with that of MYC, CSNk2A2, and CD44 from a TCGA dataset (n = 169). **(E)** The β-catenin, p33/37/41-β-catenin, GSK-3β, and P-GSK-3β protein contents in U87MG cells were assessed by western blotting. NC, cells infected with negative control lentivirus expressing Scr-shRNA; KD, cells infected with OLFML2A-shRNA-1-expressing lentivirus.

The results of previous studies have shown that the Wnt/β-catenin pathway is activated in glioma and is essential for glioma growth, proliferation, cell cycle regulation, and apoptosis (20). Importantly, the IPA data showed that Wnt/β-catenin signaling was one of the inhibited pathways enriched in the OLFML2A-associated gene signatures (**Figure 5D**). The expression of 21 molecules involved in the Wnt/β-catenin pathway was altered (**Figures 6A, B**). Subsequently, we validated the expression levels of the 21 molecules by real-time PCR and observed that the expression levels of Wnt ligands (Wnt3, Wnt5a, and Wnt5b), the transcription factor LEF1, and genes downstream of Wnt (MYC, CD44, and CSKN2A2) were decreased when OLFML2A was downregulated (**Figure 6C**). The changes in the remaining molecules were generally consistent with the gene chip results (**Figure 6C**). These data preliminarily confirmed that Wnt activity is suppressed upon OLFML2A downregulation. Furthermore, we observed that the mRNA levels of the Wnt target genes MYC, CD44, and CSKN2A2 were significantly and positively correlated with the OLFML2A mRNA levels in GBM patients by analyzing TCGA data (**Figure 6D**). Accumulating evidence has demonstrated that the regulation of β-catenin and GSK-3β phosphorylation controls Wnt/β-catenin pathway activation (20). Therefore, we subsequently assessed the phosphorylation of β-catenin and GSK-3β in U87MG cells. The results showed that the Thr41/Ser37/Ser33 phosphorylation levels of β-catenin were significantly increased upon OLFML2A downregulation (**Figure 6E**). Conversely, stabilized β-catenin levels were decreased when OLFML2A was downregulated (**Figure 6E**). Additionally, we observed that phosphorylated GSK-3β levels were significantly decreased while total GSK-3β levels were elevated upon OLFML2A downregulation (**Figure 6E**). Thus, our results indicated that high levels of OLFML2A expression may activate the Wnt/β-catenin pathway to promote cell proliferation and inhibit cell apoptosis.

### APP Acts as an Intermediate Molecule to Mediate the Downstream Regulatory Effects of OLFML2A in Glioma

**Figure 7:**
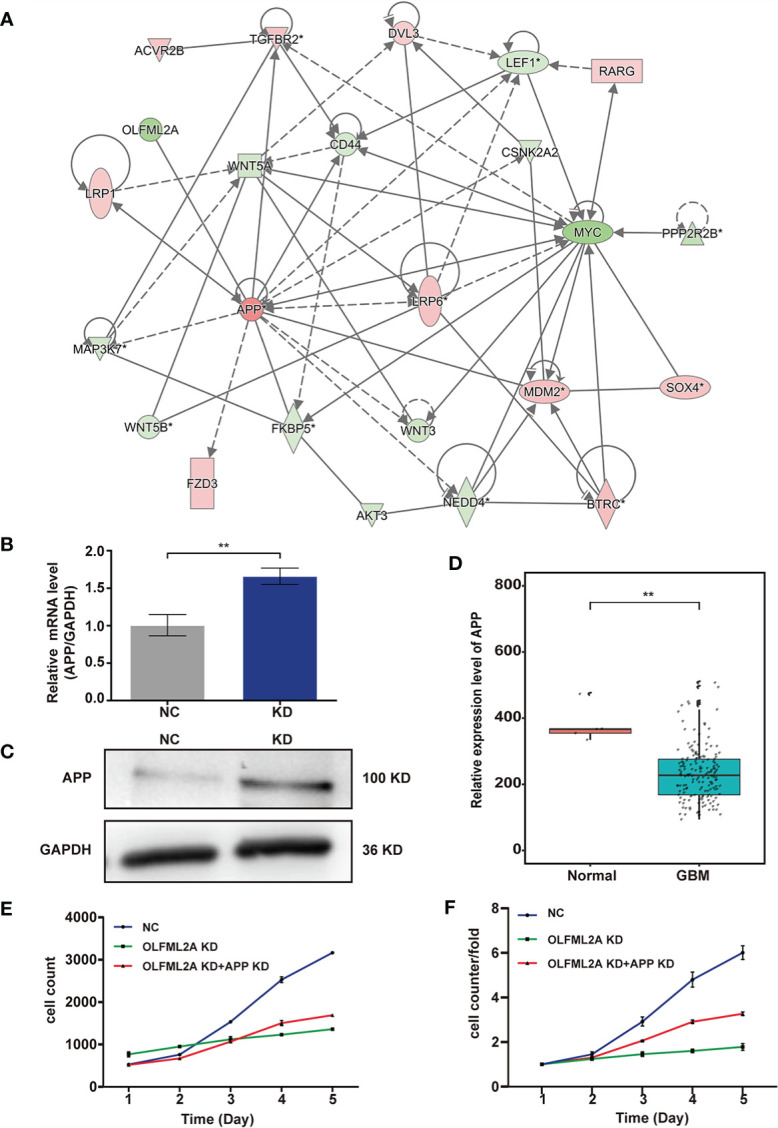
APP acts as an intermediate molecule to mediate the downstream regulatory effects of OLFML2A in glioma. **(A)** IPA-based interaction network analysis. The interaction network analysis table shows the interactions among molecules in the datasets. **(B, C)** APP expression in U87MG cells was assessed by qRT-PCR **(B)** and western blotting **(C)**. NC, cells infected with negative control lentivirus expressing Scr-shRNA; KD, cells infected with OLFML2A-shRNA-1-expressing lentivirus. **(D)** Expression profile of APP mRNA in primary GBM tissues (n=169) and normal brain tissues (n=5; Mann–Whitney U test; **P < 0.01; TCGA). **(E, F)** U87MG cell growth was measured by Celigo assays for 5 days. NC, cells infected with negative control lentivirus expressing Scr-shRNA; OLFML2A KD, cells infected with OLFML2A-shRNA-1-expressing lentivirus; OLFML2A KD+APP KD, cells infected with OLFML2A-shRNA-1 and APP-shRNA lentiviruses.

To further elucidate the regulatory mechanism of OLFML2A on the Wnt/β-catenin pathway and cell proliferation, an IPA-based interaction network analysis was performed, the results of which confirmed that APP is an important intermediate molecule that mediates the downstream regulatory effects of OLFML2A (**Figure 7A**). APP has been reported to be a negative regulator of Wnt/β-catenin signaling pathways (21). Our results showed that OLFML2A knockdown increased APP expression (**Figures 7B, C**). Furthermore, we observed that APP expression was significantly downregulated in GBM tissues compared to normal brain tissue by analyzing TCGA data (**Figure 7D**). Additionally, we demonstrated that APP downregulation partly increases the proliferation of OLFML2A-knockdown U87MG cells (**Figures 7E, F**). These data further suggested that APP is a functional regulator that mediates the downstream regulatory effects of OLFML2A through the Wnt/β-catenin pathway in glioma.

### OLFML2A Downregulation Leads to the Repression of Glioma Cell Proliferation *In Vivo*

**Figure 8:**
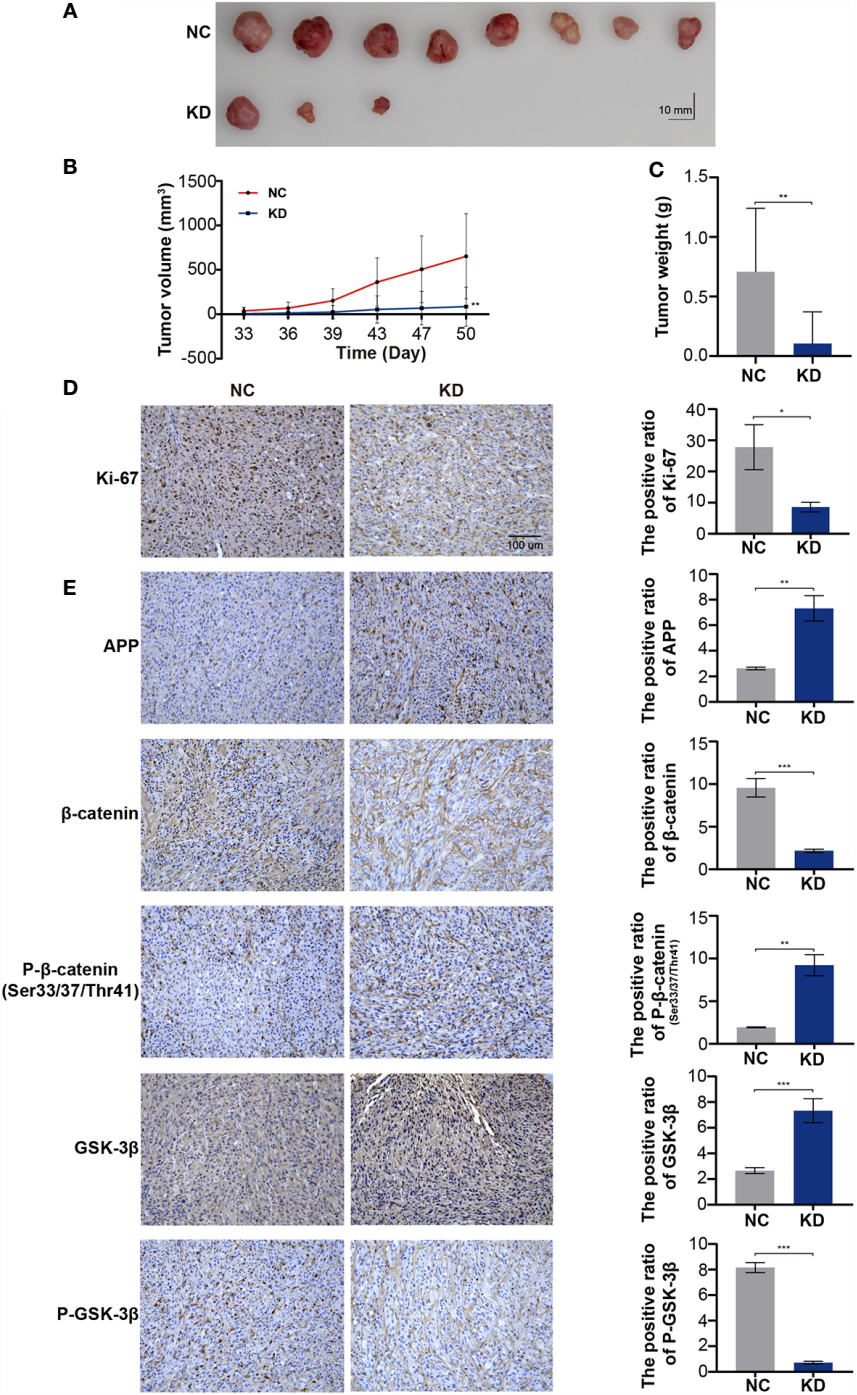
OLFML2A downregulation leads to the repression of glioma cell proliferation and inhibition of the Wnt/β-catenin signaling pathway in xenograft implantation models. **(A–C)** Subcutaneously transplanted gliomas were utilized to analyze the effect of OLFML2A on glioma *in vivo*, and tumor values were measured every 3–4 days. **(D)** Representative IHC images of Ki67 expression in two groups of subcutaneous glioma tissues; scale bar=100 µm. **(E)** APP, p33/37/41-β-catenin, β-catenin, GSK-3β, and P-GSK-3β expression in two groups of subcutaneous glioma tissues was detected by IHC. *P < 0.05, **P < 0.01, ***P < 0.001.

We used a xenograft model to verify whether OLFML2A knockdown reduces tumor growth *in vivo*. U87MG cells infected with Scr-shRNA or OLFML2A-shRNA were transplanted into the right flanks of immunocompromised nude mice, and the results showed that OLFML2A knockdown led to significant decreases in tumor volume and weight (**Figures 8A–C**) (n = 10). Moreover, the IHC results showed that OLFML2A knockdown could significantly reduce the positivity rate of Ki-67 in subcutaneous tissues (**Figure 8D**). In addition, the level of APP expression was consistent with the *in vitro* results (**Figure 8E**). Furthermore, the IHC results also indicated that β-catenin phosphorylation levels were significantly elevated while stabilized β-catenin levels were markedly decreased following OLFML2A downregulation (**Figure 8E**). These results confirmed that OLFML2A functions as an oncogene in glioma by regulating the Wnt/β-catenin pathway *via* APP *in vivo*.

**Figure 9:**
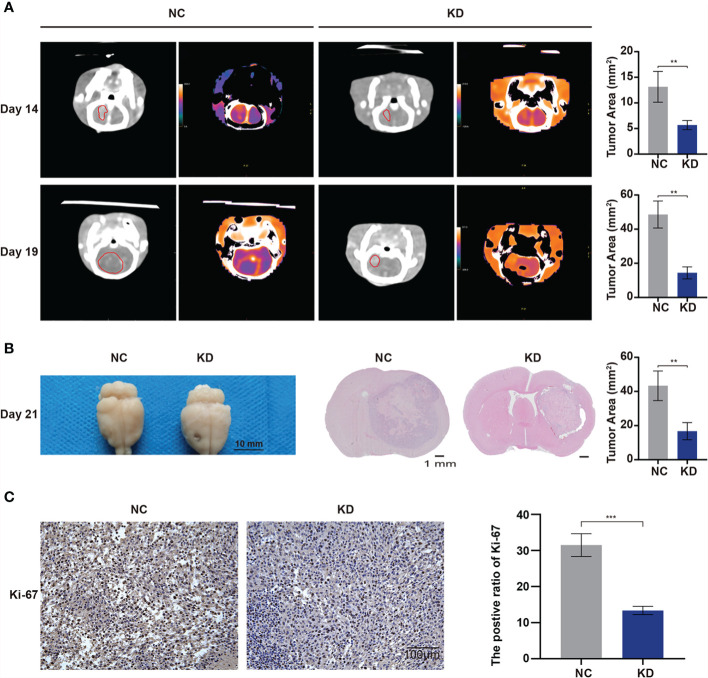
OLFML2A knockdown inhibits cell growth and proliferation in an orthotopic model. **(A)** Intracranial xenografts of gliomas were established with C6 cells after OLFML2A downregulation, and representative spectral computed tomography images show the tumor size at 14 and 19 days after transplantation in each group. **(B)** Representative gross images of gliomas after dissection from mice in each group at 21 days. **(C)** IHC staining of Ki-67 showed the proliferation state of transplanted glioma. **P < 0.01, ***P < 0.001.

To further verify the effects of OLFML2A on glioma growth in immunocompetent animals, we transplanted C6 cells transfected with Scr-shRNA or OLFML2A-shRNA into the right striatum of Wistar rats (n = 5) and observed that OLFML2A downregulation strongly suppressed the growth of intracranial glioma by CT scan on days 14 and 19 after transplantation (**Figure 9A**). Similarly, gross observations also showed that OLFML2A downregulation could significantly suppress orthotopic glioma growth (**Figure 9B**), and the positive rate of Ki-67 was also decreased after OLFML2A knockdown (**Figure 9C**).

## Discussion

Glioma is a very aggressive and heterogeneous tumor that lacks effective targeted therapies and has a relatively poor prognosis (22). Therefore, there is an unmet need to identify new targets to develop potential diagnostic and therapeutic strategies. In the present study, we first analyzed an available genomic database (TCGA) and identified OLFML2A as a putative molecule involved in glioma tumorigenesis. Our results indicated that OLFML2A is upregulated in glioma, positively correlated with tumor grade, and negatively correlated with the prognosis of glioma patients. In addition, we observed that the level of OLFML2A is correlated with glioma proliferation and apoptosis. Downregulation of OLFML2A expression dramatically suppressed the growth of glioma cells both *in vitro* and *in vivo*, and silencing OLFML2A significantly promoted the apoptosis of glioma cells. Furthermore, we confirmed that Wnt/β-catenin pathway activation is involved in OLFML2A-regulated glioma cell proliferation *in vitro* and *in vivo*. Finally, we demonstrated that APP is an important intermediate molecule that mediates the downstream regulatory effects of OLFML2A in glioma. Overall, OLFML2A was shown to primarily regulate glioblastoma through Wnt/β-catenin signaling by targeting APP. Our results suggested that OLFML2A promotes the progression of glioblastoma and may be a potential novel prognostic factor for glioblastoma. Importantly, OLFML2A may be a promising target gene for glioma treatment.

OLFML2A is a member of the OLF family (8). Interestingly, data obtained over the past several years demonstrated that OLF family proteins play important roles in neurogenesis, neural crest formation, dorsal ventral patterning, cell–cell adhesion, cell cycle regulation, and tumorigenesis and may act as modulators of critical signaling pathways (Wnt, BMP) (8). The human OLFML2A gene is located on chromosome 9q33.3 and encodes a secreted glycoprotein known as photomedin-1 (10). An understanding of the biological functions of proteins possessing the OLF domain has remained elusive (8, 10, 23). In the field of cancer research, the tumorigenic function of OLFM4 has been verified in many tumors (24, 25). Intriguingly, the tumorigenic function of OLFML2A is also gradually emerging. OLFML2A expression was shown to be negatively correlated with the biological progression and clinical features of TNBC, LIHC, and leukemia (11–13). Moreover, as previously reported, the Wnt/β-catenin pathway is a potential regulatory target of OLFML2A, through which OLFML2A regulates several malignant biological processes in LIHC (13). Consistent with previous studies, our results indicate that OLFML2A acts as an oncogene that exerts an important effect on glioma progression. Moreover, an IPA-based pathway enrichment analysis identified the Wnt/β-catenin pathway as being altered after OLFML2A downregulation.

Extensive studies have shown that abnormal activation of the Wnt signaling pathway leads to the malignant biological process of glioma, including cell proliferation, cell apoptosis, and cell invasion (26). In the classic Wnt/β-catenin pathway, Wnt ligands combine with the receptor protein frizzled (Fzd) and low-density lipoprotein receptor related protein 5/6 (LRP5/6) to form a protein complex, which then activates Dishevelled (Dvl), leading to the phosphorylation and inactivation of glycogen synthase kinase 3β (GSK-3β) and preventing stabilized β-catenin from being phosphorylated and degraded (26, 27). Stable β-catenin in the cytoplasm further enters the nucleus and binds to the T-cytokine/lymph enhancer binding factor (TCF/LEF) transcription factor, resulting in the transcription of Wnt target genes such as MYC, CD44, cyclin D1, and PPAR-δ (26, 27). In our present study, we demonstrated that the expression levels of Wnt ligands (Wnt3, Wnt5a, and Wnt5b), the transcription factor LEF1, and Wnt-downstream genes (MYC and CD44) were decreased when OLFML2A was downregulated. Furthermore, total GSK-3β levels were significantly elevated, and GSK-3β phosphorylation was decreased upon OLFML2A downregulation. Additionally, the levels of stabilized β-catenin were decreased while the Thr41/Ser37/Ser33 phosphorylation levels of β-catenin were increased when OLFML2A was downregulated. Overall, the results of our present study indicate that OLFML2A downregulation can inactivate the Wnt/β-catenin pathway to inhibit cell proliferation and promote cell apoptosis.

The results of an IPA-based interaction network analysis confirmed that APP is an important intermediate molecule that mediates the downstream regulatory effects of OLFML2A in glioma. Accumulating evidence has shown that abnormal cleavage of APP is a central process in the pathological mechanisms of Alzheimer’s disease (AD) (27–29), and the Wnt/β-catenin pathway is dysfunctional in AD brains (30–32). Furthermore, APP has been reported to downregulate β-catenin expression by increasing its degradation (32, 33), and overexpressed APP prevents β-catenin translocation into the nucleus through physical binding and precludes the transcription of Wnt target genes in AD disease models (21). These findings indicated a reciprocal regulation between APP and the Wnt/β-catenin signaling pathway. The results of the interaction network analysis were consistent with the function of APP acting as a suppressor of the Wnt/β-catenin pathway. In our present study, we observed that APP was expressed at low levels in glioma tissue. APP expression was observed to be negatively correlated with OLFML2A, and OLFML2A knockdown led to increased APP levels both *in vitro* and *in vivo*. Moreover, APP downregulation partially attenuated inhibitory effect of OLFML2A knockdown on cell proliferation. Therefore, we concluded that OLFML2A downregulation inhibits Wnt/β-catenin signaling by upregulating APP expression and reducing stabilized β-catenin levels.

## Conclusion

In summary, our results suggest that OLFML2A plays a crucial role in the proliferation of glioma and acts as an oncogene to promote the progression of glioma in humans. Mechanistically, OLFML2A knockdown inhibits the Wnt/β-catenin signaling pathway by upregulating APP expression and promoting β-catenin phosphorylation, leading to repressed MYC, CD44, and CSKN2A2 expression. Our data indicate that the oncogenic effect of OLFML2A in glioma occurs through regulation of Wnt/β-catenin signaling, which may provide a new potential therapeutic target for glioma.

## Data Availability Statement

The datasets presented in this study can be found in online repositories. The names of the repository/repositories and accession number(s) can be found in the article/**Supplementary Material**.

## Ethics Statement

The studies involving human participants were reviewed and approved by the ethics committee at the Lanzhou University Second Hospital (No. 2019A-077). The patients/participants provided their written informed consent to participate in this study. The animal study was reviewed and approved by the ethics committee at the Lanzhou University Second Hospital (D2019-117). Written informed consent was obtained from the individual(s) for the publication of any potentially identifiable images or data included in this article.

## Author Contributions

SM, LD, YP, and YZ designed the research, analyzed the data, and wrote the manuscript. SM, LD, HD, XM, XG, JL, GL, YY, YX, GY, XZ, GT, and SZ performed the experiments. All authors contributed to the article and approved the submitted version.

## Funding

The article was supported by the National Natural Science Foundation of China (8177050431), Basic Research Innovation Group Project of Gansu Province (21JR7RA432), Special fund project for doctoral training program of Lanzhou University Second Hospital (YJS-BD-31), The Hui-Chun and Tsung Dao Lee Endowment (LZU-JZH2224), and the Cuiying Scientific and Training Program for Undergraduates of Lanzhou University Second Hospital (CYXZ-10 and CYXZ2019-08).

## Conflict of Interest

The authors declare that the research was conducted in the absence of any commercial or financial relationships that could be construed as a potential conflict of interest.

## Publisher’s Note

All claims expressed in this article are solely those of the authors and do not necessarily represent those of their affiliated organizations, or those of the publisher, the editors and the reviewers. Any product that may be evaluated in this article, or claim that may be made by its manufacturer, is not guaranteed or endorsed by the publisher.

## Supplementary Material

Supplementary Table 1 Differentially expressed genes.

Supplementary Table 2 Canonical Pathways.

Supplementary Data Sheet 1 Heatmap.

## Abbreviations

OLFML2A, olfactomedin-like 2A; APP, amyloid precursor protein; TCGA, The Cancer Genome Atlas; LGG, low-grade glioma; TNBC, triple-negative breast cancer; LIHC, liver hepatocellular carcinoma; IHC, immunohistochemistry; DEGs, differentially expressed genes; IPA, ingenuity pathway analysis.
