# Supplementary figures and images for: OLFML2A Downregulation Inhibits Glioma Proliferation Through Suppression of Wnt/β-Catenin Signaling
Source: Front Oncol. 2021 Sep 28;11:717917. doi: 10.3389/fonc.2021.717917 (PMC8506028; doi:10.3389/fonc.2021.717917)

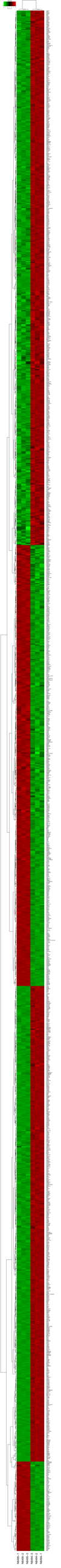

Supplement: Supplementary Data Sheet 1 — Heatmap. [file DataSheet_1.pdf]
